# Comparative Analyses of Immunosuppressive Characteristics of Bone-Marrow, Wharton’s Jelly, and Adipose Tissue-Derived Human Mesenchymal Stem Cells

**DOI:** 10.4274/tjh.2016.0171

**Published:** 2017-08-02

**Authors:** Erdal Karaöz, Pınar Çetinalp Demircan, Gülay Erman, Eda Güngörürler, Ayla Eker Sarıboyacı

**Affiliations:** 1 Liv Hospital, Center for Regenerative Medicine and Stem Cell Research and Manufacturing, İstanbul, Turkey; 2 İstinye University Faculty of Medicine, Department of Histology and Embryology, İstanbul, Turkey; 3 Onkim Stem Cell Technologies Inc., İstanbul, Turkey; 4 Eskişehir Osmangazi University Vocational Faculty of Health Services, Cellular Therapy and Stem Cell Production, Eskişehir, Turkey

**Keywords:** Immunoregulatory effect, Co-culture, mesenchymal stem cells, T cells

## Abstract

**Objective:**

Mesenchymal stem cells (MSCs), which possess immunosuppressive characteristics on induced T-cells, were shown to be applicable in prevention and treatment of graft-versus-host disease. However, knowledge of effective cell sources is still limited. In this study, MSCs from different human tissues, i.e. bone marrow (BM), Wharton’s jelly (WJ), and adipose tissue, were isolated, and the immune suppression of stimulated T cells was analyzed comparatively.

**Materials and Methods:**

MSCs were co-cultured with phytohemagglutinin-induced T-cells with co-culture techniques with and without cell-to-cell contact. After co-culture for 24 and 96 h, the proliferation rate of T cells was estimated by carboxyfluorescein succinimidyl ester and apoptosis by annexin V/PI methods. Both T cells and MSCs were analyzed with respect to gene expressions by real-time polymerase chain reaction and their specific protein levels by ELISA.

**Results:**

The results showed that all three MSC lines significantly suppressed T-cell proliferation; BM-MSCs were most effective. Similarly, T-cell apoptosis was induced most strongly by BM-MSCs in indirect culture. In T cells, the genes in NFkB and tumor necrosis factor pathways were silenced and the caspase pathway was induced after co-culture. These results were confirmed with the measurement of protein levels, like transforming growth factor β1, IL-6, interferon-γ, interleukin (IL)-2, and tumor necrosis factor-α. Additionally, IL-17A was detected in high levels in WJ-MSC co-cultures. We showed that IL-17A-producing Tregs are the key mediators in the treatment of graft-versus-host disease.

**Conclusion:**

BM-MSCs, which have been used in clinical applications for a while, showed the greatest immunosuppressive effect compared to other MSCs. However, a promising cell source could also be WJ, which is also effective in suppression with fewer ethical concerns. We described the molecular mechanism of WJ-MSCs in allogenic transplants for the first time.

## Abstract (tr)

**Amaç:**

Mezenkimal kök hücreler (MKH), uyarılmış T hücreler üzerinde sahip oldukları bağışıklık baskılayıcı özellikleri nedeniyle günümüzde graft versus host hastalığının önlenmesi veya tedavisi amacıyla kullanılmaya başlanmıştır. Kemik iliği kaynaklı MKH’lerin yanında, farklı insan kaynaklı dokulardan elde edilen MKH’lerin de benzer özelliklere sahip olduğunu bildiren raporlar yayımlanmaya başlamıştır. Bu araştırmada, günümüzde yenileyici tıp amaçlı en çok çalışılan kaynaklar olan kemik iliği (Kİ), göbek bağı (GB) ve adipoz doku kaynaklı MKH’lerin, insan uyarılmış T hücreleri üzerine olası bağışık baskılayıcı (immünsüpressif) özelliklerini karşılaştırılmalı olarak incelenmesi amaçlandı.

**Gereç ve Yöntemler:**

Uygun yöntemler kullanılarak izole edilen insan Kİ, adipoz doku- ve GB- tohemagglutinin ile uyarılmış T hücreler hücre-hücre etkileşimi veya parakrin etkiyi gözlemlenebilecek ko-kültürler tasarlandı. Yirmi dört ve 96 saatlik ko-kültürlerin ardından, T hücre çoğalımının tespiti için karboksiflüoresein süksinimidil ester ve apoptoza yönelimi tespit için ise anneksin V/PI yöntemleri kullanıldı. Hem T hücreler hem de MKH’ler gen anlatım düzeylerini değerlendirebilmek için real-time polimeraz zincir reaksiyonu ve belirli protein seviyelerin tespiti için de ELİSA yöntemleriyle analiz edildiler.

**Bulgular:**

Bulgularımız, üç farklı kaynaktan elde ettiğimiz insan MKH’lerin içinde uyarılmış T-hücreler üzerinde hem doğrudan temas yoluyla hem de parakrin etki mekanizmalarıyla hücre çoğalımını baskılamada ve apoptoza yönlendirmede en etkili Kİ-MKH’ler olduğunu göstermiştir. Bu bulgular, transforme edici büyüme faktörü (TGF)-β, interlökin (IL)-6 , interferon (IF)-γ , interlökin 2 ve tümör nekroz faktörü (TNF)-α proteinlerinin ölçümüyle de doğrulanmıştır. Bu bulgulara ek olarak GB-MKH ko-kültürlerinde IL-17A’nın arttığını ve bu sistemde IL-17A üreten Treglerin graft versus host hastalığının tedavide rol aldığını gösterdik.

**Sonuç:**

Klinikte kullanılan Kİ-MKH’lerin en etkin bağışıklık baskılayıcı etki gösterdiğini çeşitli kaynaklardan elde ettiğimiz MKH’ler ile karşılaştırarak gösterdik. Ayrıca, GB-MKH’lerin allojenik kullanımlarda altında yatan moleküler mekanizmasını ilk biz göstermiş olduk. Çalışmalarımız sonucunda kullanımında bir etik kaygı içermeyen umut vaat edici kaynak olarak, GB’yi görüyoruz.

## INTRODUCTION

The crucial role of mesenchymal stem cells (MSCs) in tissue function is widely known with their effect on the tissue components by paracrine and autocrine factors. Until the last decades, the self-renewal capacity and multilineage differentiation potency of these cells were the main focus for tissue regeneration applications. On the other hand, the chemical factors secreted by MSCs in different experimental conditions irrespective of antigen-specific or mitogenic stimulation could also affect the immune system by suppressing maturation of dendritic cells and the functions of T cells, B cells, and natural killer cells, as well as by inducing regulatory T (Treg) cells. Numerous reports showed that MSC-derived bone marrow (BM) [1,2,3], adipose tissue (AT) [2,4], Wharton’s jelly (WJ) [4,5,6], peripheral blood (PB) [6], cord blood [7], placenta [8], amniotic fluid [9], dental pulp [10,11,12], dental follicle (DF) [12], supernumerary tooth-derived stem cells [13], periodontal ligament [14], and even periapical lesions [12] suppress activated T-cell responses.

However, the molecular mechanisms underlying these effects are still unclear and need to be explored in much greater detail; they probably require both cell-to-cell contact and a variety of cytokines and soluble factors in a paracrine manner. Studies emphasized that proliferation was mainly inhibited by a paracrine effect. However, the direct co-culture of MSCs was also demonstrated to play an important role in their apoptotic effect in our recent study [11]. In this study, it was aimed both to characterize comparatively the immunosuppressive effects of MSCs derived from three different human tissues, i.e. BM, WJ, and AT, in detail and to clarify the mechanisms underlying them.

## MATERIALS AND METHODS

Human BM-, AT- and WJ-MSCs (hBM-, h-AT, and hWJ-MSCs) were isolated and then identified according to their immunophenotype by flow cytometer. MSCs were induced to differentiate in vitro. After that, in order to check the T-cell proliferation and apoptosis rate, MSCs and phytohemagglutinin (PHA)-T cells were co-cultured in both mixed lymphocyte reactions (MLR) and transwell co-cultures. Cytokine and soluble factor expressions by human MSCs (hMSCs) and PHA-T cells were analyzed by ELISA and flow cytometer to understand the paracrine effect of MSCs on PHA-T cells. After co-culture, we used the LightCycler 480 human apoptosis panel (Roche, Mannheim, Germany) to detect the apoptotic effects of MSCs on PHA-T cells and to determine which pathways are involved by real-time polymerase chain reaction (RT-PCR).

### Isolation of hBM-, hAT-, and hWJ-MSCs

BM aspirates (2-4 mL) were obtained from the iliac crest of patients (n=10, age range: 2-7 years) with suspected idiopathic thrombocytopenic purpura. Informed consent was received in accordance with the terms of the Ethics Committee of Kocaeli University. Flow cytometric analysis confirmed that the donors were healthy. The BM was diluted to 1:3 with PBS and layered over Histopaque-1077 (1.077 g/mL, Sigma-Aldrich, St. Louis, MO, USA) for gradient centrifugation. The low-density mononuclear cells were collected, washed twice with PBS, counted, and plated in tissue culture flasks at a density of 1.4x10^5^ cells/cm^2^ in low-glucose Dulbecco’s modified Eagle medium (L-DMEM) (Invitrogen/GIBCO, Grand Island, NY, USA) containing 10% fetal bovine serum (FBS), 100 IU/mL penicillin, and 100 mg/mL streptomycin. The cells were incubated at 37 °C in a humid atmosphere containing 5% CO_2_ for 3 days. On the third day, red blood cells and other non-adherent cells were removed and fresh medium was added to allow further growth. The adherent cells were grown to 70% confluence and passaged at this stage so as not to impede their proliferative capacity. Further passaging of the cells was performed by seeding 3.0x10^3^ cells/cm^2^.

Human AT tissues were obtained by lipoaspiration of subcutaneous fat of the abdominal region from donors. The donors consented to the procedure and agreed with further research on the aspirated fat in accordance with the ethical guidelines of the Kocaeli University Medical Ethics Committee. To remove blood and residues, tissue samples were washed several times with Hanks’ balanced salt solution (HBSS) (Invitrogen/GIBCO, Paisley, UK) containing 5% penicillin/streptomycin solution (Invitrogen/GIBCO, Paisley, UK) without calcium and magnesium ions. Fatty portions of the lipoaspirate samples were collected by pipette and digested in HBSS supplemented with 5 mL of 0.075% collagenase type 1 (Invitrogen/GIBCO, Grand Island, NY, USA) for 60 min in a shaking water bath at 37 °C. The digests were pipetted vigorously at 20-min intervals and dissociation was monitored microscopically. After approximately 60 min, the cell suspensions were filtered through a 70-µm cell strainer to separate single cells from debris and tissue fragments of undigested AT. Cells were re-suspended in 10 mL of L-DMEM (Invitrogen/GIBCO, Paisley, UK) supplemented with 1% penicillin/streptomycin and 10% FBS (Invitrogen/GIBCO, Paisley, UK) and were centrifuged for 8-10 min at 300 x g. Adipose cells were washed three times in culture medium. Viability of the adipose cells was determined using the trypan blue dye exclusion method and a hemocytometer. The cells isolated from eight samples were plated in separate 25-cm^2^ culture flasks containing L-DMEM with 100 U/mL penicillin (Invitrogen/GIBCO, Paisley, UK), 0.1 mg/mL streptomycin (Invitrogen/GIBCO, Paisley, UK), and 10% FBS. Three days after the initiation of culture, the medium was replaced with fresh medium, and subsequently it was replaced twice a week. When 70%-80% confluence was reached in the primary cell culture, the cells were treated with 0.25% trypsin-ethylenediaminetetraacetic acid (Invitrogen/GIBCO, Paisley, UK) for 3 min. The separated cells were collected, centrifuged, and re-plated at ratios of 1:3-1:4 for subculture.

Umbilical cords were obtained from consenting patients delivering full-term infants by Cesarean section (n=4), who faced no complications throughout pregnancy. Cords of 15 cm in length were immersed in sterile HBSS (Invitrogen/GIBCO, Paisley, UK) containing 5% penicillin/streptomycin solution (Invitrogen/GIBCO, Paisley, UK) without calcium and magnesium ions and immediately transferred to the laboratory. Arteries and vein of the cord were removed by blunt dissection, and the remaining tissue was chopped with scissors and scalpels. Enzymatic digestion was performed in HBSS supplemented with 5 mL of 0.075% collagenase type 1 (Invitrogen/GIBCO, Grand Island, NY, USA) for 30 min in a shaking water bath at 37 °C. The homogenate was centrifuged at 1500 rpm for 5 min. The cell pellet was re-suspended in the culture medium, and viable cells were counted using the trypan blue dye exclusion method and a hemocytometer.

### Immunophenotype Identification with Flow Cytometry

Undifferentiated MSCs were subjected to flow cytometry analysis to confirm that the hBM-, hAT-, and hWJ-MSCs maintained their immunophenotypic characteristics after growth in the culture. After the 3^rd^ passage, stem cells were harvested and re-suspended in their own culture medium at a concentration of 1x10^6^ cells/mL. Flow cytometry was performed using a FACSCalibur flow cytometer (Becton Dickinson, San Jose, CA, USA). The data were analyzed using CellQuest software (Becton Dickinson, San Jose, CA, USA). Debris and dead cells were gated out by forward- and side-scatter profiles. Immunophenotyping of the MSCs was performed with antibodies against the following human antigens: CD3, CD4, CD5, CD7, CD8, CD10, CD11b, CD13, CD14, CD15, CD19, CD29, CD33, CD44, CD45, CD71, CD73, CD90, CD106, CD123, CD146, CD166, HLA-DR, HLA-A, HLA-B, HLA-C, and HLA-G (Becton Dickinson, San Jose, CA, USA).

### In Vitro Differentiation

**Adipogenic Differentiation:** The cells obtained from the 3^rd^ passage were seeded at a density of 3x10^3^ cells/cm^2^ onto coverslips coated with type 1 collagen (Becton Dickinson, San Diego, CA, USA) in 6-well plates to induce adipogenic differentiation. The cells were cultured with the adipogenic medium, L-DMEM supplemented with 10% FBS, 0.5 mM isobutyl-methylxanthine (Sigma-Aldrich, St. Louis, MO, USA), 10^-6^ M dexamethasone (Sigma-Aldrich, St. Louis, MO, USA), 10 μg /mL insulin (Sigma-Aldrich, St. Louis, MO, USA), 200 µM indomethacin (Sigma-Aldrich, St. Louis, MO, USA), and 1% penicillin/streptomycin for 2 weeks. The medium was replaced twice a week. The presence of intracellular lipid droplets, which indicates adipogenic differentiation, was confirmed by Oil Red O staining.

**Osteogenic Differentiation:** Similarly, the cells obtained from the 3^rd^ passage were seeded at a density of 3x10^3^ cells/cm^2^ onto 6-well plates with coverslips coated with type 1 collagen. For osteogenic differentiation, L-DMEM was supplemented with 100 nM dexamethasone (Sigma-Aldrich, St. Louis, MO, USA), 0.05 µM ascorbate-2-phosphate (Sigma-Aldrich, St. Louis, MO, USA), 10 mM β-glycerophosphate (Sigma-Aldrich, St. Louis, MO, USA), 1% penicillin/streptomycin, and 10% FBS. The cells were incubated in this medium for 4 weeks. The medium was replaced twice a week. At the end of the 4^th^ week, osteogenic differentiation was assessed with Alizarin Red S staining.

### CD3+ T-Cell Negative Immunoselection and Stimulation

After the informed consent of 3 healthy male volunteers was received, PB was obtained with approval of the Ethics Board of the Medical Hospital of Kocaeli University. CD3+ T cells were isolated from PB using the RosetteSep T-cell enrichment negative immune selection cocktail (Stem Cell Technologies, Vancouver, BC, Canada). CD3+ T cells were stimulated with 10 µg/mL mitogen PHA (Invitrogen/GIBCO) for 24 h in RPMI-1640 medium (Invitrogen/GIBCO) containing 10% FBS, 100 U/mL penicillin, 0.1 mg/mL streptomycin, and 200 mM glutamax (Invitrogen/GIBCO). To measure CD3+ T-cell stimulation, PHA-CD3+ T cells (1x10^5^/well) were plated in triplicate onto 96-well plates and 10 µl of WST-1 (Roche Diagnostics, Mannheim, Germany) was added to each activated and non-activated (control) CD3+ T cell-containing well. The plate was incubated at 37 °C in a humid atmosphere containing 5% CO_2_ for 4 h. The optical density value of samples was measured with a microplate ELISA reader (Versamax, Sunnyvale, CA, USA) at a wavelength of 480 nm.

### Examination of CD3+ T Cells by Flow Cytometry

Flow cytometry analysis was performed as described above. Immunophenotyping of CD3+ T cell was performed with surface molecule-staining antibodies against the human antigens CD3, CD4, CD8, CD45, TCR alpha beta, and TCR gamma delta. All antibodies were obtained from BD Biosciences.

### IF Staining of CD3+ T Cells

Cells were fixed in ice-cold methanol. After permeabilizing them with 0.025% Triton X-100 (Merck, Darmstadt, Germany), the cells were incubated with 1.5% normal goat or donkey blocking serum (Santa Cruz) in PBS to suppress the nonspecific binding of immunoglobulin G. After washing, cells were incubated overnight with primary antibodies (anti-CD3, anti-CD5, anti-CD8, anti-CD23, anti-CD29, and anti-CD105). After that, cells were incubated with the appropriate FITC-labeled appropriate secondary antibodies (Santa Cruz). After washing, the cells were mounted with mounting medium containing DAPI (Santa Cruz).

### Co-culture Experiments

In MLR and transwell co-cultures, hMSCs (3x10^5^/well in 6-well plates) were plated 24 h before the addition of an equal number of PHA-CD3+ T cells to generate adherent monolayers in plates with RPMI-1640 medium. MSCs and PHA-CD3+ T cells (1:1) were co-cultured for 4 days. T cell proliferation (WST-1, CFSE), apoptosis (annexin V), cytokine and soluble factor expressions by hMSCs and PHA-CD3+ T cells, and Treg cell marker expressions were analyzed (flow cytometry, ELISA) in triplicate.

### Mixed Lymphocyte Reactions

PHA-CD3+4 T cells were plated in wells alone (for controls) or on the hDP-SC for cell-to-cell interactions. In addition to the analysis referred to above, video and photographic recordings were performed.

### Transwell Experiments

Two chambers were separated by a semipermeable membrane with a pore size of 0.4 µm (BD Biosciences). PHA-CD3+ T cells were cultured in the upper chamber of the transwell inserts. The lower chambers contained medium alone (for controls) or medium containing MSCs. In addition to the analysis referred to above, gene expression analysis was performed by real-time PCR.

### Detection of Apoptosis in Stimulated T Cells

For flow cytometry annexin V-PI labeling, cells (2x10^5^ cells) were labeled fluorescently for the detection of apoptotic and necrotic cells by adding 20 µl of binding buffer and 5 µL of annexin V-FITC (BD Biosciences) to each sample. Samples were mixed gently and incubated at room temperature in the dark for 15 min immediately. Before the analysis by flow cytometry, 2 µL of PI (1 mg/mL; BD Biosciences) was added to each sample. A minimum of 1x10^4^ cells within the gated region were analyzed. The T-cell population was identified by a combination of side-scatter and forward-scatter information.

### Cytokine Expression Profiles of PHA-CD3+T Cells and MSCs

For flow cytometry analysis, we measured the percentage of IL-6 and IL-10 expression by MSCs and IL-2, IL-6, IL-12, interferon (IFN)-γ, tumor necrosis factor (TNF)-α, CD4, CD25, and FoxP3 by PHA-CD3+ cells by flow cytometry.

For ELISA, supernatants were collected from MLR and transwell co-cultures for quantitative determination of cytokine levels, including IL-2, IL-6, IFN-γ, TNF-α, and transforming growth factor (TGF)-β. These measurements were performed by ELISA according to the manufacturer’s protocol.

For quantitative gene expression analysis by real-time PCR, total RNA from MSCs and PHA-CD3+ T cells (3x10^6^/well) was isolated using a High Pure RNA Isolation Kit (Roche Diagnostics, Germany) following the manufacturer’s instructions. After isolation, RNA quantification was measured with a Picodrop spectrophotometer (Pico100; Pico, Saffron Walden, UK). One microgram of total RNA was reverse-transcribed into cDNA using a Transcriptor High Fidelity cDNA synthesis kit (Roche Diagnostics, Germany). Equal amounts of cDNA were used for the real-time amplification of the target genes using the Universal Probe Library according to the manufacturer’s recommendations by Light Cycler 480-II (Roche Diagnostics, Germany). Primers and TaqMan® probes were designed previously and validated by the manufacturer (Roche Diagnostics, Germany). Quantification of the gene expression of IL-2, IL-6R, IL-17A, FoxP3, CD4, CD25, and IFN-γ was carried out for stimulated T cells. The expression of IL-6R, TGF-β1, IP-10, and HGF-β was measured in hMSCs. All of the quantifications were relative to the housekeeping gene hypoxanthine guanine phosphoribosyl transferase, providing a basis for the normalization of sample-to-sample differences.

### Apoptotic Gene Expressions in Activated T-cells after Co-culture

After co-culture, we used the LightCycler 480 human apoptosis panel (Roche, Mannheim, Germany) to detect the apoptotic effects of hBM-, hAT-, and hWJ-MSCs on stimulated T cells and to determine which pathways are involved by real-time PCR (qRT-PCR). Primarily, we isolated total RNA (High Pure RNA Isolation Kit, Roche) and converted it into cDNA by reverse transcriptase (Transcriptor High Fidelity cDNA Synthesis Kit, Roche). Gene expression analyses of 84 apoptosis-related genes were performed, and we determined the alterations in pro- and anti-apoptotic genes for each cell line based on a control group (stimulated CD3+ T cells).

### Statistical Analysis

All experiments were repeated at least three times. Data are reported as means ± standard deviations. All statistical analyses were performed using SPSS 10.0 (SPSS Inc., Chicago, IL, USA). Since we had one nominal variable and one measurement variable, and we wanted to compare the mean values of the measurement variable, we used Student’s t-test. Differences between the experimental and control groups were regarded as statistically significant when p<0.05.

## RESULTS

### Isolation, Culture, and Phenotype Identification of MSCs

**Figure 1:**
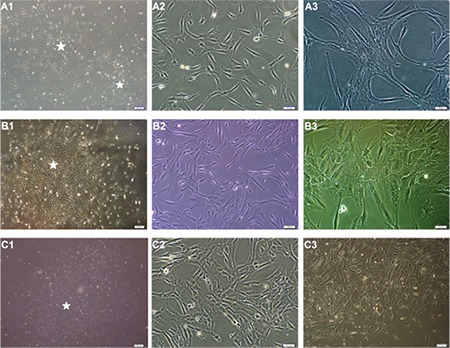
Isolated cells from human bone marrow- (A1-A3), human adipose tissue- (B1-B3), and human Wharton’s jelly- mesenchymal stem cells (MSCs) (C1-C3) distributed sparsely on the culture flasks displayed mostly fibroblast-like, spindle-shaped morphology during the early days of incubation. Small colonies (asterisks), called colony-forming units, appeared within 9-12 days (A1, A2: P0 - 9^th^ day, B1: P0 - 12^th^ day, C1: P0 - 12^th^ day). These primary cells reached monolayer confluence within 15-17 days. In the later passages, most of these MSCs exhibited large, flattened, or fibroblast-like morphology (A3, C3: P3 - 3^rd^ day, A2: P2 - 4^th^ day, C2: P1 - 6^th^ day, and B3: P3 - 7^th^ day).

**Figure 2:**
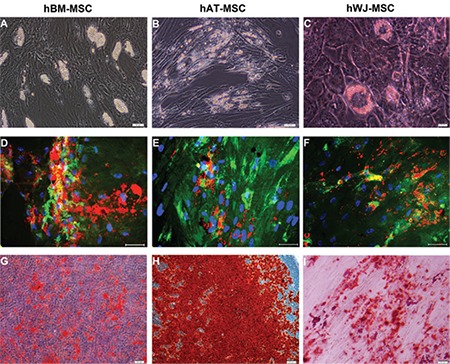
Photomicrographs of the in vitro differentiation of human bone marrow- (hBM) (A, D, G), human adipose tissue (hAT)- (B, E, H), and human Wharton’s jelly (hWJ)-mesenchymal stem cells (MSCs) (C, F, I) cultured in differentiation-inducing media as described in the materials and methods section. Phase-contrast microscopic appearances of the hBM- (A), hAT- (B), and hWJ-MSCs (C) differentiated into adipogenic lineages after 15 days and 18 days of incubation, respectively. Adipogenic differentiation was marked visually by accumulation of neutral lipid vacuoles in cultures (D, E, F) (red oil staining) (α-smooth muscle actin-green). Osteogenic differentiation of hBM- (G), hAT- (H), and hWJ-MSCs (I) after the osteogenic induction. Mineral nodules were stained positively with Alizarin Red S staining (scale bars=50 µm).

Isolated cells from hBM-MSCs (Figure 1, A1-A3), hAT-MSCs (Figure 1, B1-B3), and hWJ-MSCs (Figure 1, C1-C3) distributed sparsely on the culture flasks displayed mostly fibroblast-like, spindle-shaped morphology during the early days of incubation. Small colonies, called colony-forming units, appeared within 9-12 days (Figure 1, A1, A2, B1, and C1). These primary cells reached monolayer confluence within 15-17 days in the later passages; most of these MSCs exhibited large, flattened, or fibroblast-like morphology (Figure 1, A3, C3, A2, C2, and B3). The surface phenotypes of these cells were found to be positive for CD11b, CD13, CD29, CD44, CD73, CD90, CD146, CD166, and HLA-A, -B, and -C and negative for CD14, CD19, CD33, CD34, CD45, CD117, and HLA-DR (Table 1). We also showed that MSCs could differentiate into adipocytes and osteoblasts (Figure 2).

### Characterization and PHA Activation of CD3+ T Cells

**Figure 3:**
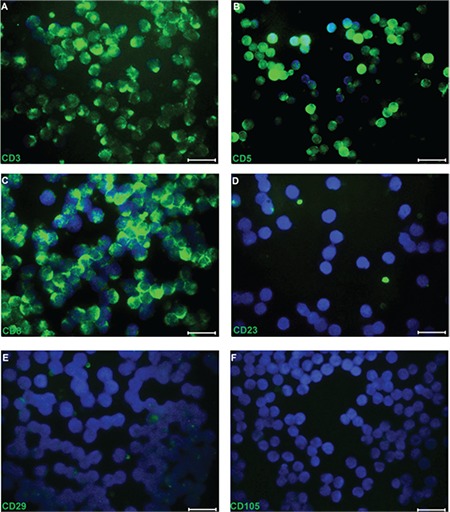
Representative panels of immunofluorescence in phytohemagglutinin (PHA)-CD3+ T cells. Fluorescence microscopy analysis of the expression of cell surface markers staining for CD3 (A), CD5 (B), and CD8 (C) was positive whereas it was negative for CD23 (D), CD29 (E), and CD105 (F) in PHA-CD3+ T cells (scale bars=50 µm).

**Figure 4:**
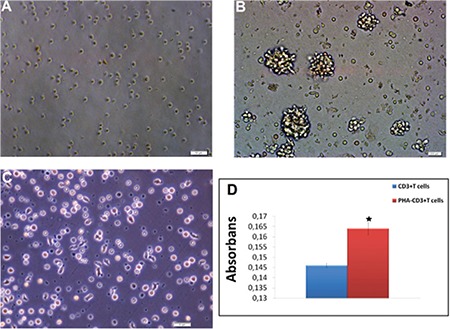
Activation of CD3+ T cells by phytohemagglutinin (PHA). Phase-contrast microscopy showing that as CD3+ T cells (A) were activated by PHA-M for 24 h, the morphologies and shapes of the cells changed, and they presented extensions (B, C). Activation of CD3+ T cells (by PHA) was determined by WST-1 (D). Increased levels of proliferation (of PHA-CD3+ T cells) were determined by WST-1 (n=3, mean ± SE, p<0.05). Activation of CD3+ T cells (by PHA) was determined by flow cytometer.

We determined that 90.67% of CD3+ T cells were isolated from PB using RosetteSep (data not shown). Immunofluorescence labeling showed that CD3+ T cells presented positive staining for CD3, CD5, CD8, and IL-12 but were negative for CD23, CD29, and CD105 (Figure 3). It was shown that in PHA-activated CD3+ T cells, their morphology changed and the cells presented extensions (Figures 4A-4C). The proliferation of PHA-CD3+ T cells showed a significant difference (p<0.05) compared with inactive CD3+ T cells (control) by the WST-1 test (Figure 4D).

### Immunoregulatory Effects of hMSCs on PHA-CD3+ T Cells

### Suppressive Effect of hMSCs on the Proliferation of PHA-CD3+ T Cells

**Figure 5:**
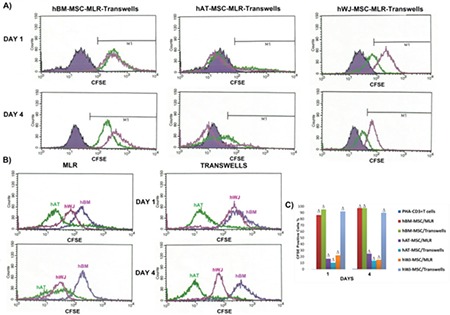
Inhibitory effect of human mesenchymal stem cells (hMSCs) on the proliferation of phytohemagglutinin (PHA)-CD3+ T cells as detected by carboxyfluorescein succinimidyl ester (CFSE). (A) Representative analysis of the altered rate of PHA-CD3+ T cells in the mixed lymphocyte reactions (MLR) and transwell experiments when co-cultured with hMSCs was performed by CFSE labeling at days 1 and 4. Solid purple histogram represents data of T cell-only culture, while green and pink peaks show the result of direct (MLR) and indirect (transwell) culture, respectively. (B) Comparative analyses of three cell cultures indicate that hBM-MSCs are the most effective cell type in the repression of induced T-cell proliferation. (C) Graphical representation of the cell proliferation in different cell cultures supports the results of previous data (n=3, mean ± standard deviation; Δ p<0.001). hBM: Human bone marrow, hAT: human adipose tissue, hWJ: human Wharton’s jelly.

The inhibitory effect of hMSCs on the proliferation of PHA-CD3+ T cells was detected by carboxyfluorescein succinimidyl ester (CFSE) (Figure 5). Comparative analyses of three cell cultures indicated that hBM-MSCs were the most effective cell type in the repression of induced T-cell proliferation. BM-MSCs also demonstrated a high level of anti-proliferative effect.

### Apoptotic Effects of hMSCs on PHA-CD3+ T Cells

**Figure 6:**
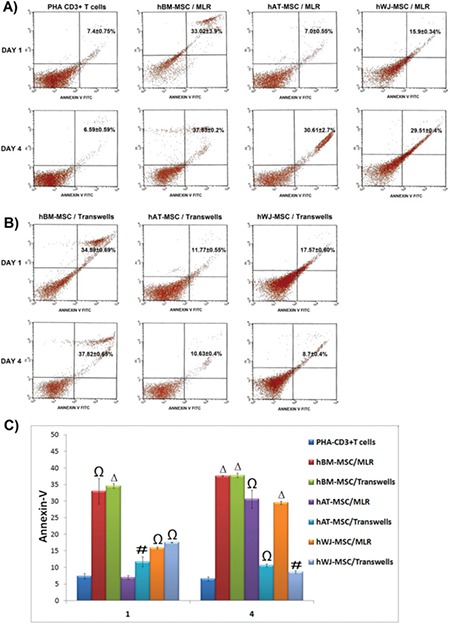
The effects of mesenchymal stem cells (MSCs) on apoptosis of phytohemagglutinin-CD3+ T cells based on annexin V-PI. Representative analysis of annexin V-PI-labeled stimulated T cells by flow cytometry in direct [mixed lymphocyte reactions (MLR)] (A) and indirect (transwell) (B) co-cultures with human MSCs on days 1 and 4 (n=3, mean ± standard deviation). The graphical representation of the data (C) points out the highest apoptotic levels of induced T cells in the co-culture with hBM-MSCs. Noticeable apoptotic levels were detected in both direct (MLR) (Ω p<0.01-1^st^ day; Δ p<0.001-4^th^ day) and indirect (transwell) (Δ p<0.001-1^st^ and 4^th^ days) co-cultures. Interestingly, the most promising results were obtained in the co-culture allowing cell-to-cell contact in both cell lines on the 4^th^ day. Weak but significant apoptotic effect was observed in indirect co-culture also in both cell lines (# p<0.05 and Ω p<0.01). hBM: Human bone marrow, hAT: human adipose tissue, hWJ: human Wharton’s jelly.

The effects of MSCs on apoptosis of PHA-CD3+ T cells based on annexin V-PI showed that the most promising results were obtained in the co-culture allowing cell-to-cell contact in hBM, hAT, and hWJ cell lines on the 4^th^ day (Figure 6).

BM-MSCs were demonstrated to have the most powerful apoptotic effect on PHA-T cells both in direct (MLR; p<0.01 - day 1; p<0.001 - day 4) and indirect (transwell) co-cultures.

### Soluble Factors Responsible for the Immunoregulatory Effects of hMSCs

**Figure 7:**
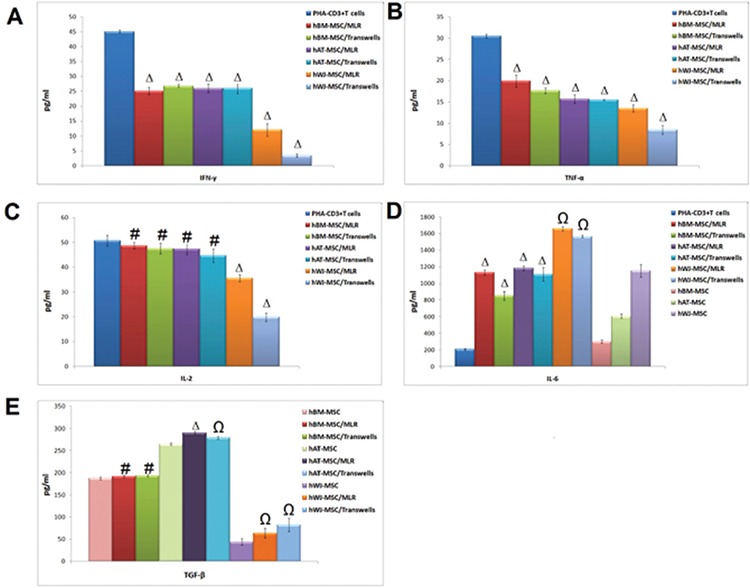
Alterations of cytokine levels in mesenchymal stem cells (MSCs) and phytohemagglutinin (PHA)-CD3+ T cells after co-culture supernatants were analyzed by ELISA. Pro-inflammatory cytokines interferon-γ, tumor necrosis factor-α, and interleukin-2 (IL-2) were inhibited significantly by MSCs in mixed lymphocyte reactions and transwell experiments on day 4. Transforming growth factor-β and IL-6 were significantly increased in both experiments on day 4 after co-culture with PHA-CD3+ T cells (n=3, mean ± standard deviation; # p<0.05; Ω p<0.01, and Δ p<0.001).

For alteration of cytokine levels in MSCs and PHA-CD3+ T cells after co-culture, supernatants were analyzed by ELISA. The pro-inflammatory cytokines IFN-γ, TNF-α, and IL-2 were significantly inhibited by MSCs in MLR and transwell experiments on day 4. There was an exception for IL-6 and TGF-β. Both were significantly increased in both experiments on day 4 after co-culture with PHA-CD3+ T cells (p<0.05, p<0.01, and p<0.001) (Figure 7).

### Immunosuppressive Effects of hMSCs on Cytokine Levels by PHA-CD3+ T Cells

**Figure 8:**
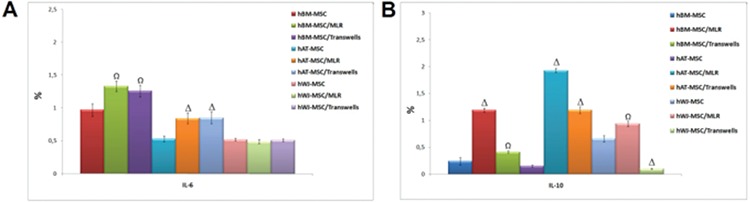
The change of cytokine levels in mesenchymal stem cells estimated by flow cytometry after co-culture experiments. The levels of interleukin (IL)-6 and IL-10 were increased in mixed lymphocyte reactions and transwell experiments after 4 days of co-culture with phytohemagglutinin-CD3+ T cells (n=3, mean ± standard deviation; Ω p<0.01, and Δ p<0.001).

The change of cytokine levels in MSCs was estimated by flow cytometry after co-culture experiments. The levels of IL-6 and IL-10 were increased in MLR and transwell experiments after 4 days of co-culture with PHA-CD3+ T cells (p<0.01 and p<0.001) (Figure 8).

**Figure 9:**
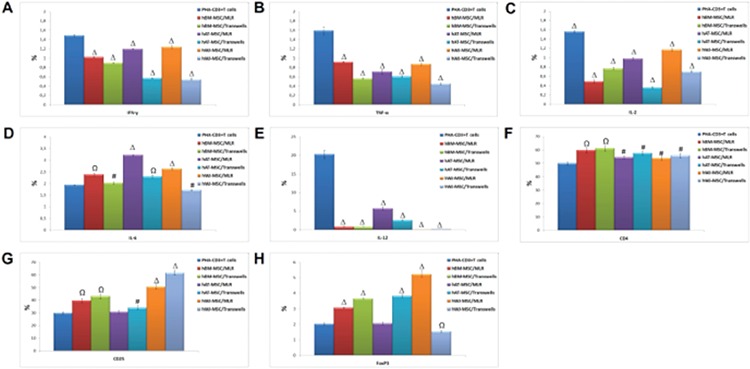
The immunoregulatory effects of mesenchymal stem cells (MSC) on the expression of pro-inflammatory cytokines (A-E) and Treg markers (F-H) in phytohemagglutinin-CD3+ T cells. The expression levels of Treg markers were significantly induced by human MSCs in mixed lymphocyte reactions and transwell experiments on day 4 (n=3, mean ± standard deviation, # p<0.05, Ω p<0.01, and Δ p<0.001).

**Figure 10:**
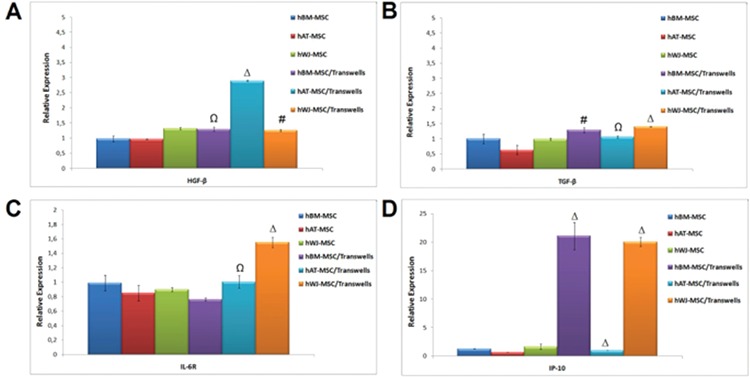
The level of cytokines and growth factors in mesenchymal stem cells was altered after co-culture (# p<0.05, Ω p<0.01, and Δ p<0.001).

**Figure 11:**
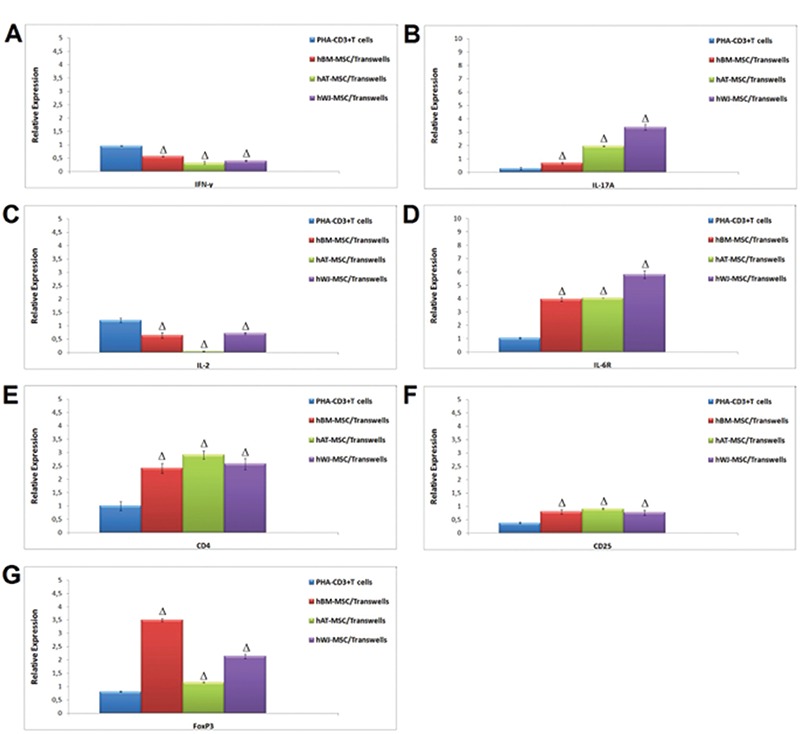
The alteration of gene expression levels of cytokines (interferon-γ, interleukin (IL)-17, IL-2, and IL-6R) and Treg markers (CD4, CD25, and FoxP3) in mesenchymal stem cells after co-culture with stimulated T cells (# p<0.05, Ω p<0.01, and Δ p<0.001).

The expression levels of Treg markers were significantly induced by hMSCs in MLR and transwell experiments on day 4 (p<0.05, p<0.01, and p<0.001) (Figure 9). By using appropriate antibodies, cells that expressed Treg markers (CD4+, CD25+, and FoxP3+) were found at a dramatically high level. These increasing levels were found to be much higher in WJ-MSCs than BM-MSCs or AT-MSCs (Figures 10 and 11).

### Apoptotic Gene Expressions in Activated T Cells after Co-culture

**Figure 12:**
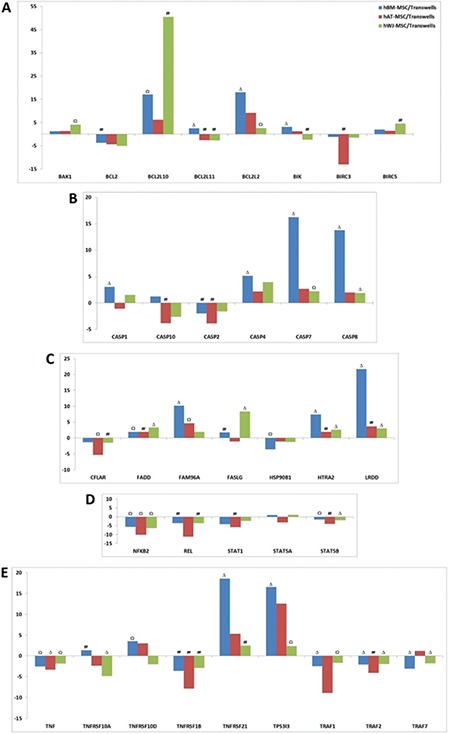
Estimation of apoptotic gene expressions in activated T cells after co-culture with human bone marrow-, human adipose tissue-, and human Wharton’s jelly-mesenchymal stem cells (MSCs). The downregulation of JAK-STAT-related pathways was observed in cells after co-culture (A, D, E). Upstream of the caspase-linked apoptotic pathway and related genes, HTRA2, FADD, and FASLG, were induced in T cells by co-culture with human MSCs (B, C).

When gene expressions of T cells after co-culturing with MSCs were compared with induced CD3+ T cells (Figures 12A-12E), the expression of survival pathways and proliferation-related genes (NFKB2, REL, RELB, STAT5A-B, etc.) were significantly reduced (Figure 12D). Expression levels of caspase family member genes responsible for apoptosis induction, CASP1, CASP4, CASP7, and CASP8, were increased (Figure 12B). These genes were especially significantly increased in hBM-MSCs. Caspase activity-related gene HTRA2 was significantly increased in hBM- and hWJ-MSCs (Figure 12C). Expressions of apoptosis death pathway genes FADD and FASL were significantly increased, similar to one of the death domain-including genes, LRDD (Figure 12C). We determined that TNF expressions were irregular for TNF pathway-related genes, which is important for apoptosis regulation. Gene expression of the TNFRSF21 gene, known as death receptor 6 (DR6), was ~18-fold increased when comparing BM with the control (Figure 12E). Curiously, apoptosis inhibitor BIRC3 (c-IAP1) expression that includes the CARD (caspase activation and recruitment domain) region was increased in hAT-MSCs.

Gene expressions of BAK1 and BIK, which are pro-apoptotic members of the BCL-2 family, were upregulated and anti-apoptotic member BCL-2 was downregulated. Gene expression results show that the paracrine effects of MSCs induce apoptosis in activated T cells. During this process, a decrease was observed in expression of the BCL2 and BIRC3 anti-apoptotic genes, and an increase was observed in expression of the HRK and LRDD pro-apoptotic genes and tumor-suppressor genes TP53I3 and PTEN. The NFKB2, REL, RELB, SOCS2, STAT1, STAT5A, and STAT5B genes and the TNF, TNFRSF1B, and TRAF1 genes were determined to have a role in the inhibition of NFKB and TNF pathways, respectively.

## DISCUSSION

Due to the immunoregulatory properties of MSCs, they have begun to be used for prevention of graft-versus-host disease (GvHD) and treatment in allogenic transplantations. In this study, different MSC sources, including BM-MSCs that are widely used in clinical applications and are the most effective cells, WJ-MSCs that are readily available tissue from umbilical cords without any ethical concerns, and AT-MSCs that are also an easily accessible source for stem cells for regenerative medicine, have been comparatively analyzed for immunosuppressive effects on PHA-T cells. Direct co-culture (MLR) was applied in order to evaluate cell-to-cell contact and indirect co-culture (transwell) was applied for paracrine effects.

Hepatocyte growth factor-β (HGF) and TGF-β are the main mediators secreted by MSCs and they act as immunosuppressors [1]. Furthermore, IL-6 has been defined to act as both a pro-inflammatory and an anti-inflammatory cytokine that inhibits T-cell proliferation and stimulates apoptosis [15,16], which may involve induction of the classical “anti-inflammatory” cytokine IL-10 [17]. In our study, we confirmed IL-10 expression by flow cytometer, and based on our data, the expression level of IL-10 was significantly higher after direct contact with MSCs and PHA-T cells. We also found that productions of TGF-β1 and IL-6 were increased significantly compared to PHA-T cells. The anti-proliferative effect of MSCs is most probably a result of increased production of TGF-β1 and IL-6 in the co-culture with MSCs.

We analyzed the hallmark cytokines produced in these co-cultures by ELISA in order to evaluate the effect of MSCs on development. We found that both IFN-γ and TNF-a production was inhibited in the presence of MSCs.

In several studies, MSCs have been demonstrated to inhibit the expression of T helper-1 pro-inflammatory cytokines [18]. In this study, we demonstrated that gene expressions of IL-2, IL-12, and IFN-γ were inhibited in MLR and transwell co-culture systems. In support of this, TGF-β1 and IL-10 inhibit T-cell proliferation by suppressing IL-2, IFN-γ, and TNF-α production.

We tried to confirm our suggestion with the KEGG pathway analysis tool. We showed that IL-6, IL-2, IL-12, IFN-γ, and TNF-α are key mediators for GvHD and allograft rejection (Picture 1).

IP-10 is a member of the ELR family of α-chemokines [19,20]. In recent years, this chemokine was found to be important for suppression of PHA-T cells by MSCs. In our study, the gene expression level of IP-10 was increased after the indirect co-culture of BM-MSCs and WJ-MSCs. This finding seems compatible with the CFSE experiments, in which we detected an anti-proliferation effect of MSCs on PHA-T cells.

In programmed cell death, the caspase pathway plays an important role. For instance, CASP1 plays a central role in the initiation of caspase family activation. The upregulation of this gene further triggers the level of other caspases. Finally, the programmed cell death process continues irreversibly with increased levels of CASP7. In our study, some major effectors of apoptosis such as CASP1, CASP4, CASP7, and CASP8 were upregulated in PHA-T cells. This effect was higher when BM-MSCs were co-cultured with PHA-T cells.

Consistent with these results, caspase activity-related pro-apoptotic genes HTRA2 and LRDD (also known as PIDD) were significantly upregulated in both BM-MSC and WJ-MSC co-cultures on PHA-T cells (Figure 12C).

Gene expression of the TNFRSF21 gene that is known as death receptor 6 (DR6) was ~18-fold increased when comparing the co-cultures with BM-MSCs and PHA-T cells (Figure 12E).

In addition, we found that expressions of survival pathways and proliferation-related genes NFKB2, REL, and STAT5A-B were significantly downregulated (Figure 12D).

We also analyzed differentially expressed genes according to our apoptosis panel and GvHD with the Phenolyzer phenotype-based gene analyzer tool [21]. This tool compares 4 different databases and gives seed gene and predicted gene lists according to disease or phenotype. IL-10 and TNF were given as seed genes, which means they might be the main cause of GvHD on a molecular scale. Analysis results were consistent with our findings: we demonstrated that immunosuppressive cytokines were increased in co-cultures and in the tool’s predictions TGF-β and IL-6 were related to GvHD. Apoptotic genes were also upregulated and again this tool confirmed our results, showing the relation of the caspase family, HTRA2, LRDD, and TNFRSF21, with GvHD (Picture 2).

One of the most important findings of our study is the correlation of IL-17A level and Tregs. IL-17A, a pleiotropic pro-inflammatory cytokine produced by Th17 T-helper cells, was initially described as an important mediator for neutrophil induction and maturation during inflammatory responses [22]. We detected the increasing of CD4+ CD25+ FoxP3+ Tregs by flow cytometer parallel to significant upregulation of IL-17A in gene expression in PHA-T cells after indirect co-cultures with WJ-MSCs. In other words, we think that IL-17A is required by CD4+ CD25+ Tregs to sustain an immunosuppressive response. IL-17A-producing T-cells may open up a new path for prevention and treatment of GvHD.

Our data have indicated additional important findings for WJ-MSCs. Pro-apoptotic genes such as BCL2L10, BAK1, and BIK were upregulated and expressions of apoptosis death pathway genes FADD and FASL significantly increased in co-culture of PHA-T cells and WJ-MSCs. In addition to these results, anti-apoptotic genes such as BCL-2 and BIRC3 were downregulated in the PHA-T cells after co-culture.

We identified genes that were differentially expressed in WJ-MSC co-cultures in order to confirm our findings. We detected that apoptotic genes BAK1, BIK FADD, and FSLG and anti-apoptotic gene BCL2 are related to GvHD. Additionally, we analyzed IL-17, which we think has potential for treatment of GvHD, and FoxP3, one of the Treg markers. We demonstrated that IL-17 is related to disease and FoxP3 is directly in interaction with both seed genes for GvHD (Picture 2).

## CONCLUSION

In conclusion, our results have demonstrated that BM-MSCs, which are still the most preferable source in clinics, are highly effective. Having said that, WJ-MSCs, readily available tissue from WJ without any ethical concerns, could be an alternative source for clinical use; they might especially be a very effective therapeutic for preventing allograft rejection and in GvHD treatment. WJ is being used for allogenic transplants in the clinic. We demonstrate the molecular mechanism underlying the cause of treatment. We have determined that AT-MSCs, an easily accessible source for stem cells for rejuvenating and regenerative medicine, have the weakest immunosuppressive activity when compared to BM-MSCs and WJ-MSCs. Additionally, MSCs show their anti-proliferative effects by the paracrine mechanism on PHA-T cells, although cell-to-cell contact is needed to show their apoptotic effect.

## Figures and Tables

**Table 1:**
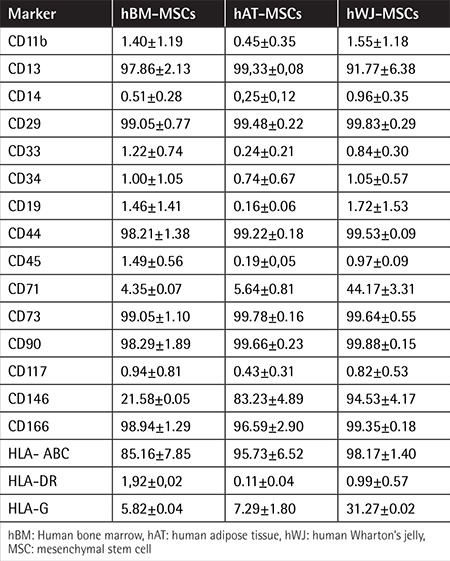
Immunophenotypic properties of hBM-, hAT-, and hWJ-MSCs using flow cytometry.

**Picture 1:**
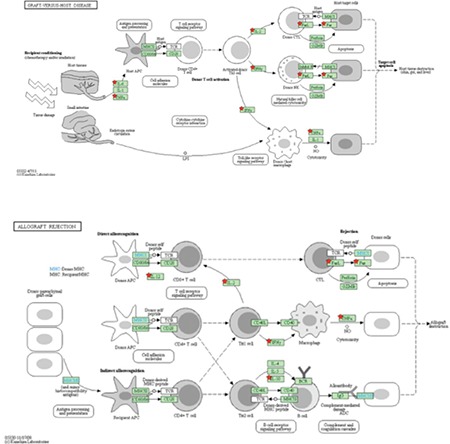
Key mediators of graft-versus-host disease and allograft rejection are confirmed by KEGG pathway analysis. IFN: Interferon, IL: interleukin, IgG: immunoglobulin g, TCR: T cell receptor MHC: major histocompatibility complex, TNF: tumor necrosis factor.

**Picture 2:**
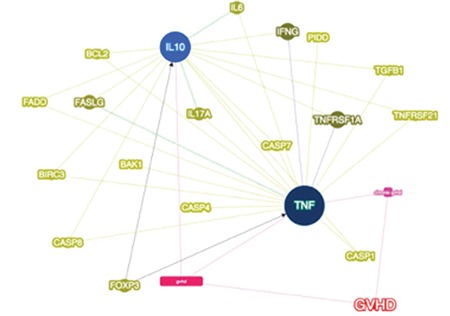
Tumor necrosis factor and interleukin (IL)-10 are represented as seed genes for graft-versus-host disease (GvDH). The correlation of differentially expressed genes in the apoptosis panel was described. The importance of IL-17A and FOXP3 for GvDH was confirmed by four different databases [21].
